# The Application of Deep Learning for the Evaluation of User Interfaces

**DOI:** 10.3390/s22239336

**Published:** 2022-11-30

**Authors:** Ana Keselj, Mario Milicevic, Krunoslav Zubrinic, Zeljka Car

**Affiliations:** 1 Department of Electrical Engineering and Computing, University of Dubrovnik, 20000 Dubrovnik, Croatia; 2 Faculty of Electrical Engineering and Computing, University of Zagreb, 10000 Zagreb, Croatia

**Keywords:** machine learning model, user interface design, automatic evaluation, design analysis, deep learning

## Abstract

In this study, we tested the ability of a machine-learning model (ML) to evaluate different user interface designs within the defined boundaries of some given software. Our approach used ML to automatically evaluate existing and new web application designs and provide developers and designers with a benchmark for choosing the most user-friendly and effective design. The model is also useful for any other software in which the user has different options to choose from or where choice depends on user knowledge, such as quizzes in e-learning. The model can rank accessible designs and evaluate the accessibility of new designs. We used an ensemble model with a custom multi-channel convolutional neural network (CNN) and an ensemble model with a standard architecture with multiple versions of down-sampled input images and compared the results. We also describe our data preparation process. The results of our research show that ML algorithms can estimate the future performance of completely new user interfaces within the given elements of user interface design, especially for color/contrast and font/layout.

## 1. Introduction

A virtual learning environment is an online software platform that provides students with digital solutions that enrich learning, enable access to learning content regardless of time and location, and facilitate knowledge-sharing through online communication [1]. In addition to traditional and virtual learning environments, a combined (hybrid) form of learning is also described in the literature. The combined form of learning considers the best of both basic forms, e.g., by integrating technologies such as augmented reality into instruction that physically takes place in the classroom [2]. In all forms of learning, students should learn together, regardless of their difficulties and differences. Therefore, it is very important to apply the principles of inclusion in education. Inclusion is based on the social model of disability, which emphasizes how people with disabilities can be included in all aspects of life, including education, employment, etc., by adapting the environment and providing support [3]. Teaching students with learning disabilities presents unique challenges. Children with disabilities often have limited attention spans, making it difficult for them to stay engaged with a task for an extended period of time.

One of the most important features of the learning experience in traditional and virtual learning environments is the ability to interact with software solutions [4]. Traditional two-dimensional user interfaces of computer systems are familiar to most users. Interaction with these interfaces mainly occurs through input devices such as a mouse or keyboard, using a screen as an output device. Touchscreens can be used as both an input and an output device. The advancement of computer hardware and software has led to the development of user interfaces and software solutions for which traditional input devices such as a keyboard and mouse often cannot be used [5]. In addition to the ability to interact, one of the most important aspects of educational applications is accessibility.

If we consider user interfaces as a spectrum of customization possibilities, on one side of the spectrum, there are interfaces that can be customized by the user to increase the usability and efficiency of user interaction. On the other side of the spectrum are intelligent user interfaces. According to [6], intelligent user interfaces (IUI) aim to improve human–computer interaction, especially the user experience and/or the usability of user interfaces using artificial intelligence (AI). The paper also contains a thorough survey of efforts to evaluate user experience (UX) and the usability of IUIs over the last decade. It identifies research gaps in IUI evaluation and examines IUI research, systematic literature reviews, and systematic mapping studies—for example, intelligent, context-sensitive, and multimodal user interfaces, adaptive user interfaces, intelligent human–computer interaction, and adaptable and adaptive user interfaces.

In the context of this spectrum of adaptation possibilities, our work lies somewhere in the middle. The goal of this study was to test the hypothesis that a machine-learning model (ML) can evaluate the future performance of a user interface in terms of user response and that such a model is capable of evaluating different user interface designs within the defined boundaries of some given software. We investigated whether there is a way to automatically evaluate existing and new web application designs and give developers and designers a benchmark for choosing the most user-friendly and effective designs for their software. This principle can apply to both 2D and 3D contexts, with the 3D context to be verified in ongoing research. We found no comparable method for ranking potential application designs in the available literature.

In the user interaction format used to test our hypotheses, different user options are available for selection, which further depend on the user’s knowledge. In other words, the selection of some correct system parameter is a result of a particular decision made by the user based on their cognitive reasoning. This interaction format is very common in quizzes used specifically in virtual learning environments. The quiz is a tool for independent learning; one study has shown that engineering students find quizzes motivating and encourage regular learning [7], while voluntary use of online quizzes, as well as the results obtained, is a useful general indicator of student performance in the medical field [8]. Authors of another paper presented the results of a pilot project using adaptive quizzes in a fully online unit delivered by an Australian higher education provider [9]. The project results suggest that adaptive quizzes contribute to student motivation and engagement and that students believe that adaptive quizzes support their learning. Therefore, the quiz case study offers a useful example of an application with diverse and wide-ranging uses both in virtual learning environments and beyond.

The results of the research presented in this paper show that our ML model can analyze a interface design in its entirety (in our specific example, this includes contrast, colors, arrangement of elements, font, and text size). The peculiarity of the method also lies in the preparation of the data for the learning of the neural network. To avoid bias, learning data were prepared by removing extremes and responses from real users that did not make sense in terms of user interaction with the application.

Accessibility is an additional practical implementation for the results we obtained. The model can learn to rank accessible designs and evaluate the accessibility of new designs in terms of preferred relationships between interface elements, layouts, and colors. In this case, the learning data should be different, but the proposed approach based on ML can still be applied.

## 2. Related Work

In addition to rapid incremental progress in web-based applications, end-user satisfaction is critical to successful adoption [10]. Ease of use, perceived usefulness, and appropriateness of user interface adaptation are the three most frequently rated variables. Questionnaires appear to be the most popular method, followed by interviews and data log analysis. Van Velsen et al. [11] noted that the quality of most questionnaires is questionable, and reporting on interviews and think-aloud protocols is perceived to be superficial. The reports that were found lacked empirical value. Therefore, the authors proposed an iterative design process for adaptive and adaptable systems.

Miraz et al. [12] presented a review of research on universal usability, plasticity of user interface design, and the development of interfaces with universal usability, focusing on the fundamentals of adaptive (AUI) or intelligent user interfaces (IUI) in terms of three core areas: artificial intelligence (AI), user modeling (UM), and human–computer interaction (HCI). The paper emphasizes that more research is needed to determine the benefits and effectiveness of IUI compared to AUI. It also discusses the question of placing adaptive control of the interface under the system or the user, with application to e-learning being a priority: the use of machine intelligence to achieve appropriate learning, ideally reinforced by “game-like interaction”, was considered desirable. Performance evaluations of user interface plasticity have shown that the use of dynamic techniques can improve the user experience to a much greater extent than simpler approaches, although optimizing the tradeoffs between usability parameters requires further attention.

In one study, AlRawi [13] used usability metrics to evaluate the relation between web application usability and end-user performance. This relationship was investigated using observations and user feedback sessions. The results suggest a possible relationship between system usability and end-user performance in terms of effectiveness and satisfaction.

There are several approaches to designing a user-friendly interactive website. One approach comprises standard evaluation methods, such as the method presented in [14]. In this paper, Kaur and Sharma investigated the usability problems of selected popular web applications based on various parameters, using the traditional observational methods of usability testing. Wang [15] analyzed the priorities in interface design that are important for elderly people. Using a semi-structured questionnaire, they surveyed the needs of elderly internet users and obtained several indicators that describe their specific needs from web interfaces. Using hierarchical analysis, they calculated the weight of those indicators. Based on the results, they made suggestions to improve the accessibility of the interface for older people.

Malik et al. concluded that most researchers are interested in introducing a variety of UI-based models to improve the UI designs of web-based applications [10]. In one example, user classification and modeling are presented for improving the design flow of web sites [16], while another describes standardized user interfaces for RIAs (rich internet applications) that can improve usability [17].

There are also more innovative approaches, such as an approach that uses an evolutionary algorithm for automatically generating website designs by treating parameters of functionality, layout, and visual appearance as variables [18]. A chromosome structure has been developed that allows for representing website characteristics in terms of the three aspects mentioned above and facilitates the application of genetic operators [19]. Real-time usage mining (RUM) exploits the rich information provided by client logs to support the construction of adaptive web applications. Rich information about the behavior of users browsing a web application can be used to adapt the user interface in real-time to improve the user experience. This approach also offers support for detecting problematic users and profiling users based on the detection of behavioral patterns.

In this way, the research problems in this area of interactive software system development relate not only to the evaluation of user satisfaction, but also to the measurement of system responsiveness, efficiency, and accessibility [10].

One of the most coveted and valuable applications of ML in UX design is its ability to provide users with a new level of personalization [20]. ML algorithms that learn from usability data sources can improve the user experience [21], such as by implementing and testing a system for designing creative web elements using an interactive genetic algorithm in which voting-based feedback from the learning mechanism enables the system to adopt quality measures for visual aesthetics [22]. One systematic review of the literature that was conducted to identify the challenges UX designers face when incorporating ML into their design process contains recommendations based on its findings [20]. In one study, ML-design tools based on UX could use formal models to optimize graphical user interface layouts to meet objective performance criteria [23], while another used ML to automatically vectorize existing digital GUI designs (using computer vision) to quickly apply them to new projects [24]. ML can also facilitate the quantifiable evaluation of given GUIs by using a set of user perception and attention models [25].

In one paper, a thorough review of the last decade’s efforts in IUIs, UX, and usability evaluation is presented [6], identifying research gaps in IUI evaluation. In existing IUI-related research, systematic literature reviews and systematic mapping studies have investigated the following user interfaces: (i) intelligent, context-sensitive, and multimodal user interfaces, (ii) adaptive user interfaces, (iii) intelligent human–computer interaction, and (iv) adaptive and adaptable user interfaces. The authors concluded that the most used AI methods are deep-learning algorithms (widely used in various types of recognition) and instance-based algorithms, commonly used with the aim of human/body motion recognition, human activity, gesture, depression, and behavior recognition. The use of artificial neural networks was also identified, as well as their successful use in gesture and emotion recognition.

## 3. Data Collection and Preparation Phase

The goal of this study was to determine whether deep-learning methods are able to evaluate future performance of a user interface in which respondents solve simple mathematical tasks. This was accomplished by recording user accuracy and solution time as they used a web application that was developed for this research. The technologies chosen were HTML, SCSS, JavaScript, and PHP 7.0. Our idea was to develop an application that randomly generates its layout, background, and font color, font family, and font size from predefined classes described in SCSS. We planned for at least 300 respondents completing the questionnaire to obtain a sufficient amount of data for machine-learning needs. Undergraduate and graduate students from the University of Zagreb and the University of Dubrovnik were selected as the main target group. Survey data were collected via web application, optimized for use on mobile devices, that respondents accessed.

The application consists of 15 randomly generated questions with four offered answers, only one of which is correct. The 15 questions are divided into three cycles of five questions each. At the beginning of each cycle, a new design is presented to the user.

The questions were elementary mathematical equations to avoid a possible bias due to the knowledge of the participants. To encourage participants to read the entire question text, new question text was generated from a predefined set of questions.

For processing purposes, the application records information about the user interface that was randomly assigned to the participant (layout, combination of colors, contrast, and type and size of the font), the question, the answers, the respondent’s recorded answer, and the time the respondent spent answering. Response time was measured from the moment at which the interface of the specific question was fully loaded and displayed to the respondent until the moment at which the respondent answered the question and the application began loading the next question. Based on the data collected on the appearance of the layout and the question asked, images of the user interface shown to the respondent were created and stored for deep learning purposes.

**Figure 1:**
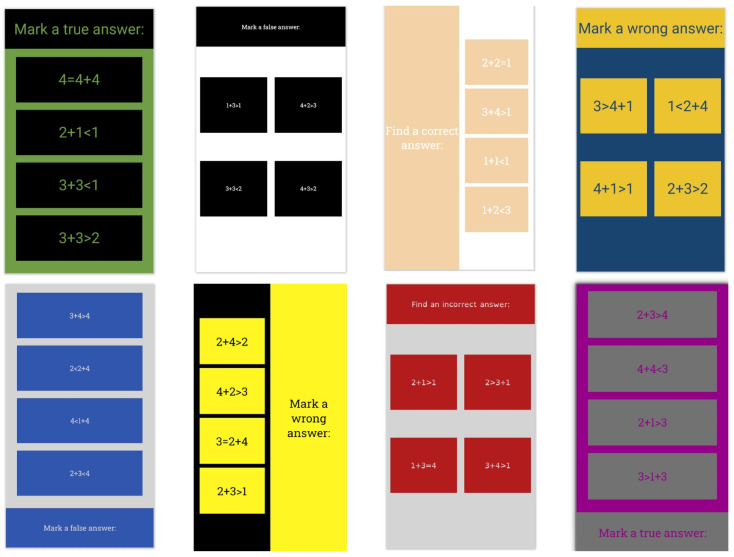
Some combinations of layouts, colors, and fonts.

The application was designed to resemble the classic applications for quizzes that are used on online platforms for e-learning. There are six different layouts of elements. Their HTML classes, with descriptions, can be found in Appendix A Table A1. A total of 18 different combinations of font and background colors were used; their RGB hex codes and contrast ratios can be found in Appendix A Table A2. The background and font colors were chosen according to the methodology for the development of an accessible website presented in [26], which states that the preferred contrast between background and text is 7:1, and the minimum contrast is 4.5: 1. This methodology provides a recommendation of eight color combinations. Used font types can be found in Appendix A Table A3. In addition to sans-serif and serif fonts, the dyslexic-friendly font OpenDyslexic (OpenDyslexic font, https://opendyslexic.org/, accessed on 20 October 2022) was also used. Chosen font sizes were 16 px, 18 px, 27 px, and 36 px. Some combinations of layouts, colors, and fonts can be seen in Figure 1. Combinations of the mentioned layouts, colors, fonts, and font sizes were used for training and validation dataset.

**Figure 2:**
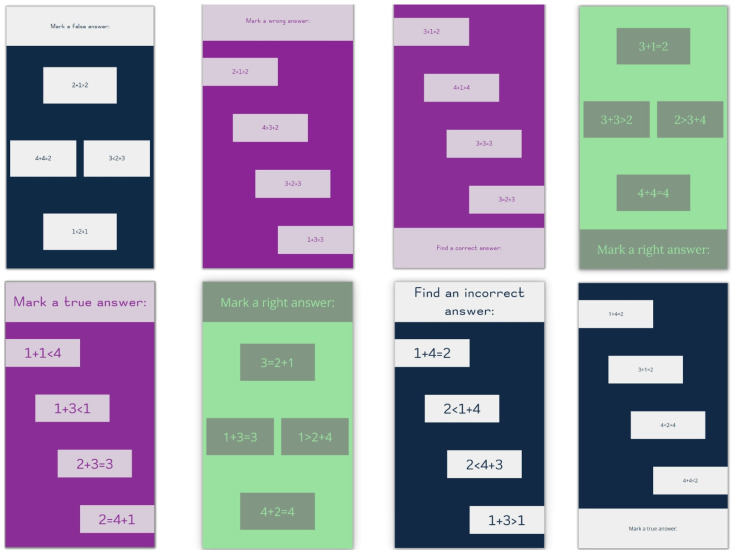
Examples of new interfaces used for testing.

As usual, part of the basic dataset was used during the initial testing phase. However, to test the real capabilities of the models, an additional test dataset (Figure 2) was prepared with previously unseen combinations of elements, including four new layouts (Table A4). The colors used and their contrast ratio used in the generated test dataset are presented in Table 1. Font families used for testing were Lora as the serif font, Open Sans as the sans-serif font and Omotype (Omotype font, https://omotype.com/, accessed on 20 October 2022) as the dyslexic-friendly font.

To process the images of the interface, the text of the question was replaced by the letter “a” to prevent the deep learning algorithm from basing its inference on the specific text of the question. The replacement with the letter “a” was done because it is highly expressive and it carries substantial font family character, as discussed in [27].

## 4. Materials and Methods

To prove the hypothesis that CNNs can evaluate the effectiveness of a user interface, we tested a number of diverse architectures: vanilla CNNs, general-purpose networks modified for regression tasks such as VGG19 (Visual Geometry Group) [28], InceptionResNetV2 [29], Xception [30], and ResNet50 [31], and deep ensemble models for regression.

CNN training was implemented with the Keras [32] and TensorFlow [33] deep-learning frameworks. We used a workstation equipped with an AMD Ryzen Threadripper 3960X CPU and NVIDIA GeForce RTX 3090 with 24 GB memory and the Linux Ubuntu 20.04 OS.

Early stopping and a model checkpoint were used for callback function. Early stopping interrupts the training process if there is no improvement of the validation loss after a defined number of epochs. The model checkpoint is used to save the best model if and once the validation loss decreases.

During the experiments, it was necessary to pay attention to the following important facts:

- Input data are non-square images
- The possibilities of using augmentation are very limited, since any mirroring or rotation of the image, or change in the brightness and contrast, significantly changes the appearance and efficiency of the interface
- Input data carry important information at different levels of detail. This means that attention should be paid to details captured by both high and low spatial frequencies. For example, the size or shape of the letters of the used font can be equally important information, as well as the position of the question in relation to the position of the offered answers.

Two solutions have been proposed for the high and low spatial frequency problem. The first solution is an ensemble of multiple custom CNNs that use different Conv2D kernel size and stride values. The second solution is based on an ensemble that uses a standard architecture and multiple versions of down-sampled input images.

Ensemble methods can improve the predictive and generalization performance of a single model by mixing predictions from several models [34]. Deep ensemble learning models [35] combine the advantages of both the deep-learning models and ensemble learning, so that the final model has better generalization performance.

### 4.1. Ensemble of Custom CNNs

**Figure 3:**
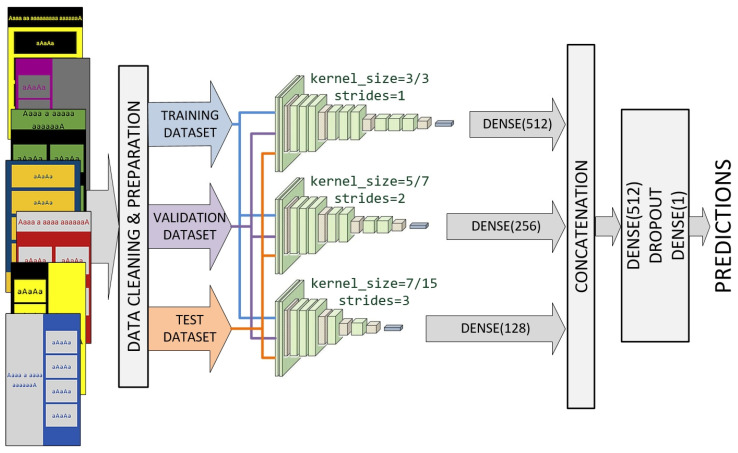
Ensemble model involving a multi-channel custom CNN.

We designed an ensemble model involving a multichannel custom CNN (Figure 3). Each channel consists of the input layer that defines the various sizes of input images, focusing on a particular scale. All channels share the standard CNN architecture in the transfer mode with the same set of filter parameters. The outputs from the three channels are concatenated and processed by dropout and dense layers.

Each channel was inspired by VGG architecture and consists of a combination of depth Conv2D, BatchNormalization, MaxPooling2D, and Dropout layers of different depth. The first channel uses kernel sizes of (3, 3) and strides of (1, 1) for all convolutional layers. The second channel uses kernel sizes of (7, 7) and strides of (2, 2) for the initial three convolutional layers. Kernel sizes of (5, 5) and strides of (1, 1) are used for the remaining convolutional layers of the second channel. Finally, the third channel uses kernel sizes of (15, 15) and strides of (3, 3) for the initial three convolutional layers. Kernel sizes of (7, 7) and strides of (1, 1) were used for the remaining convolutional layers of the third channel. The mentioned values were reached after numerous experiments.

As shown in Figure 3, channels that use larger values for kernel sizes and strides will have fewer layers. Outputs from all three channels are concatenated into a single vector and process by a Dense–Dropout–Dense combination of layers.

### 4.2. Xception-Based Ensemble

**Figure 4:**
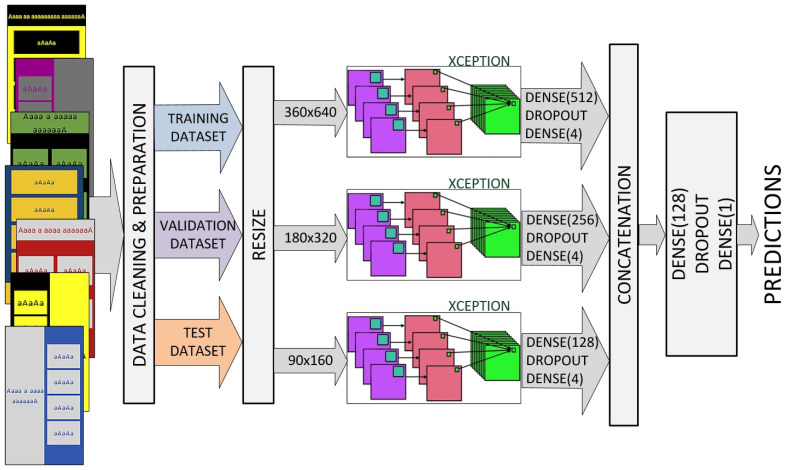
Xception-based ensemble with multiple versions of down-sampled input images.

We also designed an ensemble model that uses a standard architecture and multiple versions of down-sampled input images. Several standard architectures were tested; the best results were achieved using the Xception model (Figure 4).

Each channel has an input layer that defines the various sizes of input images (360 × 640, 180 × 320, and 90 × 160, pixels respectively). We replaced the standard Xception top layer with a Dense–Dropout–Dense combination of layers. Outputs from all three channels are concatenated into a single vector and processed by a second Dense–Dropout–Dense combination of layers.

Both the transfer learning and learning from scratch approaches were analyzed. However, in this case, ImageNet pre-trained features do not contribute to the learning process as in some other experiments, due to the large differences between the source and target task/domain, as well as the importance of the spatial arrangement of elements.

Data-cleaning and the preparation process are presented in the next section.

## 5. Results and Discussion

### 5.1. Statistical Analysis of Participant Responses

**Figure 5:**
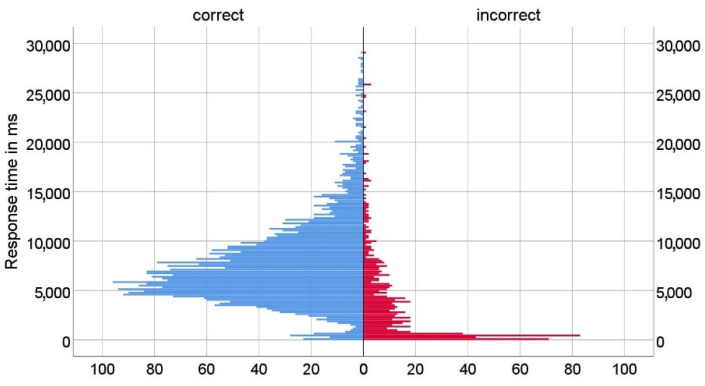
Distribution of response time (correct and incorrect answers).

In total, 338 participants (mostly students of the University of Zagreb and the University of Dubrovnik) took part in the research. The gender and age of participants were not systematically assessed, as participants were selected based on their matriculation in Bachelor- and Master-level degree programs. As some participants did not answer all questions, we collected 4448 answers in total. First, we analyzed all participant responses (correct and incorrect). The distributions of response times of correct and incorrect answers are shown in Figure 5. The results show that both distributions are positively skewed, and a disproportionate number of incorrect answers were answered in a time of less than 0.5 s.

Since the goal of the study was to investigate the impact of different user interfaces in tasks where accuracy is important, we excluded from the set all data where respondents did not choose the correct answer (17.18% of answers), whereupon 3684 correct answers remained in the set. In the remainder of the research, we processed only the data where respondents had answered correctly.

**Figure 6:**
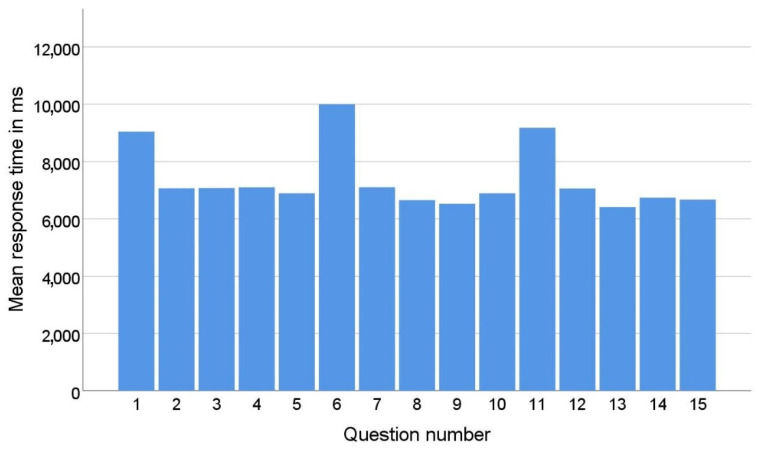
Mean response time by question number.

In the quiz, respondents were shown a new interface appearance with the first question of each series (1st, 6th, and 11th questions). The appearance and settings were different from the interfaces the respondent had seen before in the application. While answering the questions in a series, the respondent became accustomed to the new look of the interface. As a result, the average response time to the first question in each series is significantly longer than the average response time to the other questions in that series, as shown in Figure 6. This phenomenon can be explained by the theory of universal design [36]. Namely, the principle of universal design, which refers to simplicity and intuitiveness, posits that a design should be stable and predictable. This means that once a user gets used to a certain layout and interaction flow when working with the software, they should not experience unexpected design changes, as this leads to confusion. If changes are unavoidable, as in the case of online stores when the user is redirected to the payment pages, these changes should be announced in advance.

To eliminate the effects of the respondent’s adaptation to the new user interface, these questions were excluded from the training and testing set.

Since the questions are simple and the questionnaire was designed so that there is only one correct answer, when the respondent recognizes the correct answer, they do not have to read the other answers that are below it.

In this environment, the response time when the answer is in the first position could be much shorter than the response time when the correct answer is in the later positions; e.g., if the correct answer is in the last position, the respondent must read the question and all four answers to get to it. To eliminate the influence of the position of the correct answer on the response time, the response time was normalized using Equation (1).

(1) $$\mathrm{normalized}\;\mathrm{response}\;\mathrm{time}=\frac{\mathrm{response}\;\mathrm{time}}{\mathrm{position}\;\mathrm{of}\;\mathrm{answer}+1}$$

**Figure 7:**
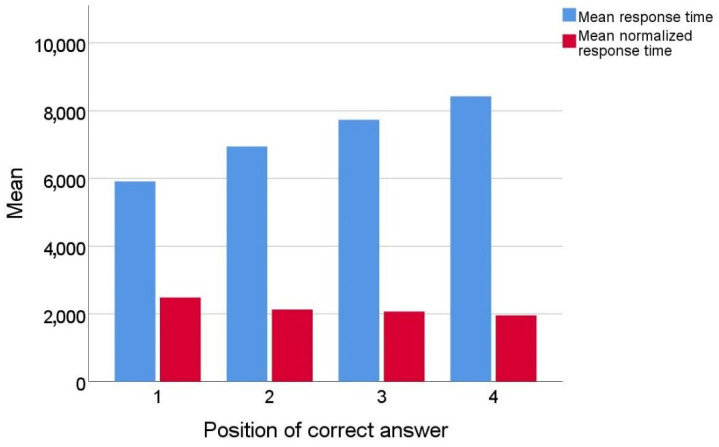
Mean response times (original and normalized) by position of correct answer.

The graph in Figure 7 shows mean response times by position of correct answer after normalization.

During the normalization process, real response time was divided by the answer position increased by 1 (time to read the question). The assumption for such normalization was the fact that most people read text intensively when they need to answer a question [37]. For example, to answer a question with the correct answer in position 2, they must read at least the question and two answers. This normalization did not completely eliminate the influence of the position of the correct answer on the response time, but the response time was significantly reduced (the difference between the largest and smallest average time before normalization was 3.39 s and after normalization was 1.04 s). Part of the difference that occurred when answering the last question could be due to the fact that some of the respondents who had not found an answer in the three previous positions chose the last answer without reading the text of that answer.

Further analysis revealed two problematic groups of response times. The first group included very short times, by which the respondent would not have been able to read the question and at least one answer. The second group included outliers in the form of very long times, for which we assumed that something prevented the respondent from answering or that the application or mobile device had performance problems while answering.

Such problematic responses accounted for about 2% of all responses. Since they could have a negative impact on the research, we decided to exclude from the set all responses for which the normalized times were shorter than 0.5 s and longer than 5 s. After this exclusion, 98% of the correct answers remained in the set, or 2632 answers in total.

**Figure 8:**
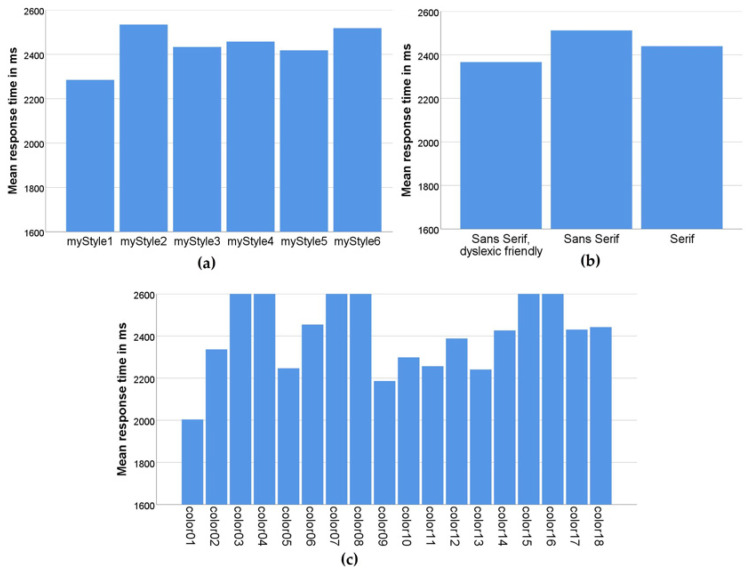
Mean response times (**a**) by layout style; (**b**) by font type; (**c**) by color combination.

Figure 8 shows mean response times by the three different criteria that formed different user interfaces in the application: layout, font, and color combination.

Figure 8a shows how efficient participants were in solving tasks using different layouts. From this graph, we can see that participants performed best with the myStyle1 layout, in which the question is at the top of the screen and the answers are arranged in a column below the question [38]. The myStyle5 layout, in which the question is in the same place but the answers are arranged in two rows (zigzag layout) was second in terms of efficiency. We assume that in this layout, changing the reading direction of the answer from horizontal to vertical saved time in retrieving the information.

Furthermore, based on the effect of font on the task-solving efficiency, as shown in Figure 8b, the dyslexic-friendly font had a positive effect.

The average reaction time of the participants by different color combinations is shown in Figure 8c. The best results were obtained with high-contrast combinations (yellow–black, color01; black–white, color05; black–yellow, color09; blue–yellow, color10; green–black, color11; white–black, color13). Most of the efficient color combinations (color01, color09, color10, and color11) are combinations from the methodology created in a previous study on the use of efficient color and contrast combinations on the web [39]. Apart from that, good results were obtained when using interfaces with high-contrast monochrome black and white (color5) and white and black (color13) combinations.

### 5.2. Evaluation of Effectiveness Using CNN Models

As described in the previous section, the process of data-cleaning and preparation resulted in the elimination of wrong answers, outliers, and answers resulting from user adaptation to the new interface. Normalization was also conducted to reduce the influence of the position (1)–(4) where the correct answer is found. Ultimately, the corrected dataset contained 2632 samples.

The available data were pseudo randomly divided into three datasets: 263 images (10%) were set aside as the test dataset, while the rest was divided into a training dataset of 2106 images (80%) and a validation dataset of 263 images (10%). The division was made in such a way that the exact same interface (taking all elements into account) was not represented in multiple datasets.

The performance of the proposed models was evaluated using mean absolute error (MAE) and root mean square error (RMSE) metrics, expressed by Equations (2) and (3) (Table 2):

(2) $$\mathrm{MAE}=\frac{1}{N}\overset{N}{\underset{i=1}{\sum }}\left|y_{i}-{\hat{y}}_{i}\right|$$

(3) $$\mathrm{MSE}=\sqrt{\frac{1}{N}\overset{N}{\underset{i=1}{\sum }}{\left(y_{i}-{\hat{y}}_{i}\right)}^{2}}$$

where *y_i_* is the ground-truth value, ${\hat{y}}_{i}$ is the predicted data and *N* is the number of testing samples.

It should be noted that in this case, some standard metrics were not suitable for the analysis of predictions. For example, the coefficient of determination R2 does not provide a comparison of different algorithms. The reason lies in the fact that for one interface, the entire response time range will be obtained (distributed mostly according to the normal distribution), and the prediction will actually be reduced to the mean value.

The results show that two proposed ensemble models achieved better performance than individual models. For the user interfaces represented in the test dataset, with the best model applied, the range of user response time values was between 1366 and 2011 ms.

It is important to note that the expected response time for a specific user interface, for example, of 1550 ms, does not mean that all users will achieve the same or a similar time. The actual response time will depend on many additional parameters, including the user’s cognitive abilities or their current mood. However, if the experiment is repeated a sufficient number of times, a mean response time close to the predicted value can be expected for a particular interface.

Thus, perhaps the main benefit of the proposed approach is the possibility of ranking interface proposals. An additional experiment was conducted in which additional interfaces were made with elements that were not used before. This refers to the arrangement of objects, used colors, fonts, etc.

**Figure 9:**
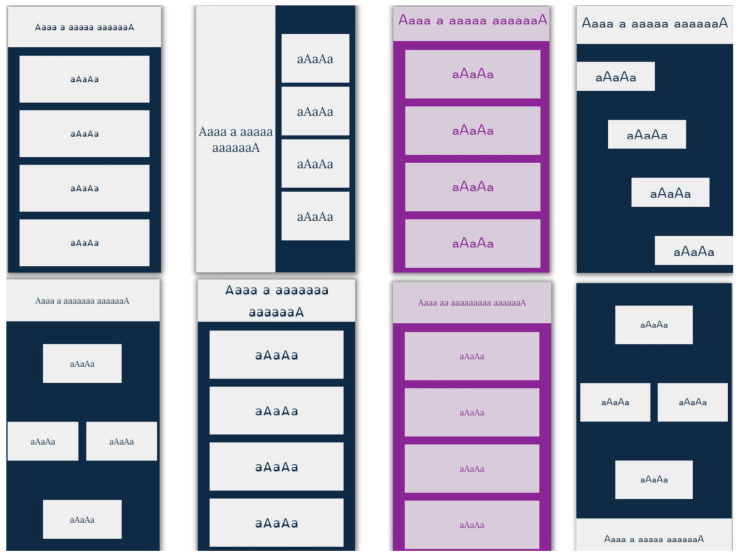
The 8 best-ranked user interfaces.

**Figure 10:**
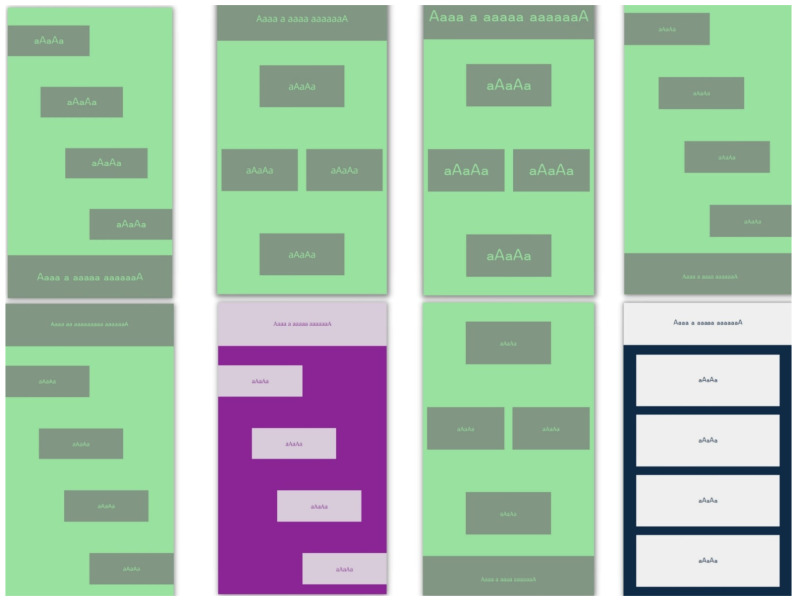
The 8 worst-ranked user interfaces.

The best model was applied to the additional test data to rank the interfaces according to the expected mean response time. Examples of the best and worst-ranked interfaces are shown in Figure 9 and Figure 10. In Figure 9, the images are ordered starting from the best response time, while in Figure 10, the images are ordered starting from the worst response time.

Analysis of the ranked interfaces reveals that deep-learning models can recognize the essential attributes of an interface and their influence on its future efficiency.

## 6. Conclusions

In this study, we successfully tested our hypothesis that our ML model can evaluate the future performance of completely new UI in terms of user response and that it is able to evaluate different designs of UI within the defined boundaries of some given software. In the user interaction format used to test the research hypotheses, different user options are available for selection that depend on user knowledge, which is common in software environments such as e-learning quizzes and others as well. Combinations of design layouts, colors, fonts, and font sizes were used in the training dataset. Model evaluation was performed by combining subject metrics from 300 research participants and the objective metrics related to user response times and answer correctness. A multi-channel ensemble model for CNN was proposed and used, and our results suggest that this approach can be applied to the classification of various UI designs. To confirm our initial hypothesis, an additional dataset with entirely new and previously unseen combinations of elements, colors, and fonts was constructed for an additional testing phase.

Our plan for further research includes extending the ML model with UI designs of different sizes/resolutions and with different interface elements and their layout, with all interfaces having previously known usability ratings. Based on this, we will test the hypothesis that ML models can evaluate a completely unknown interface. Such capability would be useful so that design is not just left to the creativity and good practices of designers and developers, but also to the formal definition and practical application of objective knowledge about UI usability, accessibility, and/or performance, thereby increasing user satisfaction and software efficiency.

## Figures and Tables

**Table 1:** Font and background colors used for testing, along with their contrast ratio and corresponding HTML class.

Class	Background Color	Font Color	Contrast Ratio
color1	#EDEEEE	#112A46	12.52:1
color2	#D8CCDA	#8B2596	4.84:1
color3	#829684	#99E19F	2.05:1

**Table 2:** The prediction performance of the models on the test datasets (ms).

Model	MAE	RMSE
vanilla CNN	789	1094
VGG-19 (scratch)	814	1155
VGG-19 (transfer)	819	1171
Xception (scratch)	785	1067
Xception (transfer)	795	1113
Ensemble of custom CNNs	741	978
Xception-based ensemble	734	954

## Data Availability

Not applicable.

## Appendix A

**Table A1:** HTML classes used for layout with description of positions of question and answers, used for purpose of training and validation.

Class	Position of Question	Position of Answers	Answers Layout
myStyle1	Top of the page	Below question	4 answers in the form of a list
myStyle2	Bottom of the page	Above question	4 answers in the form of a list
myStyle3	Right side of the page	Left of the question	4 answers in the form of a list
myStyle4	Left side of the page	Right of the question	4 answers in the form of a list
myStyle5	Top of the page	Below the question	4 answers grouped by 2 in 2 lines
myStyle6	Bottom of the page	Above question	4 answers grouped by 2 in 2 lines

**Table A2:** Font and background colors used for application, along with their contrast ratio and corresponding HTML class.

Class	Background Color	Font Color	Contrast Ratio
color1	#FEFF26	#000000	19.54:1
color2	#ECC431	#1A4571	5.86:1
color3	#000000	#709E44	3.15:1
color4	#000000	#C72E2B	5.44:1
color5	#000000	#FFFFFF	21:1
color6	#D4D4D4	#3255AE	4.65:1
color7	#F2D2A6	#FFFFFF	1.44:1
color8	#727272	#940088	1.66:1
color9	#000000	#FEFF26	19.54:1
color10	#1A4571	#ECC431	5.86:1
color11	#709E44	#000000	3.15:1
color12	#C72E2B	#000000	5.44:1
color13	#FFFFFF	#000000	21:1
color14	#3255AE	#D4D4D4	4.65:1
color15	#FFFFFF	#F2D2A6	1.44:1
color16	#940088	#727272	1.66:1
color17	#D4D4D4	#B41D1D	4.52:1
color18	#B41D1D	#D4D4D4	4.52:1

**Table A3:** Font family from application with corresponding HTML class.

Font Name	Font Family
OpenDyslexic	Sans serif, dyslexic-friendly
Roboto	Sans serif
Roboto Slab	Serif

**Table A4:** HTML classes used for layout with description of positions of question and answers, used for purpose of test.

Class	Position of Question	Position of Answers	Answers Layout
myStyle1	Top of the page	Below question	4 answers in diagonal form
myStyle2	Bottom of the page	Above question	4 answers in diagonal form
myStyle3	Top of the page	Below question	4 answers in rhombus form
myStyle4	Bottom of the page	Above question	4 answers in rhombus form
